# Inhibition Effects of a Synthesized Novel 4-Aminoantipyrine Derivative on the Corrosion of Mild Steel in Hydrochloric Acid Solution together with Quantum Chemical Studies

**DOI:** 10.3390/ijms140611915

**Published:** 2013-06-04

**Authors:** Sutiana Junaedi, Ahmed A. Al-Amiery, Abdulhadi Kadihum, Abdul Amir H. Kadhum, Abu Bakar Mohamad

**Affiliations:** 1 Department of Chemical and Process Engineering, Faculty of Engineering and Built Environment, Universiti Kebangsaan Malaysia, Bangi, Selangor 43600, Malaysia; E-Mails: sutianajnd10@gmail.com (S.J.); amir@eng.ukm.my (A.A.H.K.); drab@eng.ukm.my (A.B.M.); 2 Applied Chemistry Division, Applied Science Department, University of Technology, Baghdad 10066, Iraq; 3 Applied Physics Division, Applied Science Department, University of Technology, Baghdad 10066, Iraq; E-Mail: abdulhadikadhim@yahoo.com

**Keywords:** corrosion inhibitor, electrochemical impedance spectroscopy, 2-methylbenzaldehyde

## Abstract

1,5-Dimethyl-4-((2-methylbenzylidene)amino)-2-phenyl-1*H*-pyrazol-3(2*H*)-one (DMPO) was synthesized to be evaluated as a corrosion inhibitor. The corrosion inhibitory effects of DMPO on mild steel in 1.0 M HCl were investigated using electrochemical impedance spectroscopy (EIS), potentiodynamic polarization, open circuit potential (OCP) and electrochemical frequency modulation (EFM). The results showed that DMPO inhibited mild steel corrosion in acid solution and indicated that the inhibition efficiency increased with increasing inhibitor concentration. Changes in the impedance parameters suggested an adsorption of DMPO onto the mild steel surface, leading to the formation of protective films. The novel synthesized corrosion inhibitor was characterized using UV-Vis, FT-IR and NMR spectral analyses. Electronic properties such as highest occupied molecular orbital energy, lowest unoccupied molecular orbital energy (EHOMO and ELUMO, respectively) and dipole moment (μ) were calculated and discussed. The results showed that the corrosion inhibition efficiency increased with an increase in the EHOMO values but with a decrease in the ELUMO value.

## 1. Introduction

**Scheme 1:**
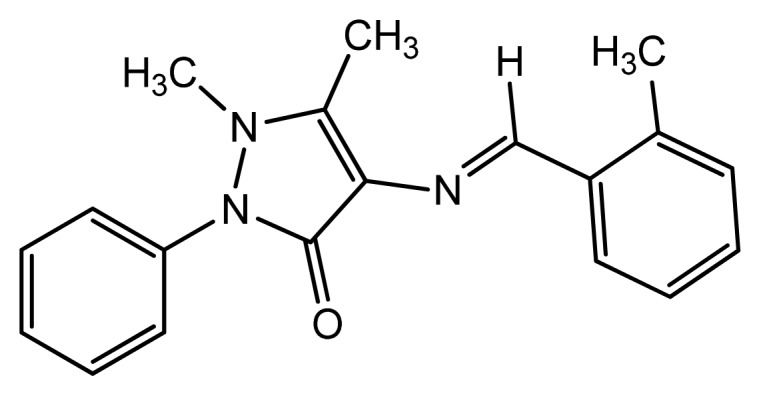
Chemical structure of 1,5-dimethyl-4-((2-methylbenzylidene)amino)-2-phenyl- 1*H*-pyrazol-3(2*H*)-one (DMPO).

Corrosion inhibitors are of considerable practical importance, as they are extensively employed in reducing metallic waste during production and in minimizing the risk of material failure, both of which can result in the sudden shut-down of industrial processes, which in turn leads to added costs [1]. It is also important to use corrosion inhibitors to prevent metal dissolution and minimize acid consumption [2–4]. The majority of well-known acid inhibitors are organic compounds that contain nitrogen, sulfur and oxygen atoms. The inhibitory action exercised by organic compounds on the dissolution of metallic species is normally related to adsorption interactions between the inhibitors and the metal surface [5,6]. The planarity (p) and lone pairs of electrons present on N, O and S atoms are important structural features that control the adsorption of these molecules onto the surface of the metal. The purpose of this work was to verify the previously established results on the corrosion inhibition effect of various Schiff bases on mild steel in acidic media [7]. Many researchers have reported that the inhibition effect depends mainly on some physicochemical and electronic properties of the organic inhibitor related to its functional groups, steric effects, electronic density of donor atoms and orbital character of electrons donor [8]. Schiff bases are organic compounds that have the general formula R–C=N–R-, where R and R- are aryl, alkyl or heterocyclic groups. Schiff bases are formed by the condensation reaction of a primary amine and a ketone or aldehyde and are potential corrosion inhibitors. The greatest advantage of many Schiff base compounds is that they can be conveniently and easily synthesized from relatively cheap materials. Due to the presence of the imine group (–C=N–) and electronegative nitrogen, sulfur and/or oxygen atoms in the molecule, Schiff bases have been reported to be effective inhibitors for the corrosion of steel in acid media by several authors [9–12]. Conversely, the surface state and excess charge of the metal have also been reported to affect the adsorption behavior of inhibitor molecules onto the metal surface [13]. Generally, the tendency to form a stronger coordination bond, consequently resulting in high inhibition efficiency, increases in the order of O < N < S < P [14]. As a continuation of previous studies [15–20], we focused on the synthesis of new heterocyclic compounds as novel organic corrosion inhibitors. Herein, we report the synthesis of 1,5-dimethyl-4-((2-methylbenzylidene)amino)-2-phenyl-1*H*-pyrazol-3(2*H*)-one, DMPO, and chemical structure elucidation using spectroscopic techniques (*i.e.*, UV-Vis, IR and NMR). Recent studies have shown that organic compounds containing polar functional groups are quite efficient in minimizing the effect of corrosion in addition to heterocyclic compounds containing polar groups and π-electrons. The molecular design of the DMPO molecule is based on the fact that 4-aminoantipyrine consists of amine, methylamine, carbonyl and π-electron bonds, which would effectively contribute towards the inhibition of mild steel corrosion in acidic media. Moreover, Schiff bases containing imine groups would contribute more effectively to the inhibition of corrosion of mild steel in acid medium. The resonance effect of DMPO increases its inhibition activity. Structural parameters, including the frontier molecular orbital (MO) energies, specifically HOMO (highest occupied molecular orbital) and LUMO (lowest unoccupied molecular orbital) energies and dipole moments, were calculated and correlated with corrosion inhibition efficiencies. The calculated values of inhibition efficiencies (IE_cal_%) were obtained from a quantitative structure-activity relationship (QSAR) and were subsequently correlated with the experimentally obtained values of inhibition efficiencies (IE_exp_%). The proposed structure of the synthesized novel corrosion inhibitor is shown in Scheme 1.

## 2. Results and Discussion

### 2.1. Chemistry

**Scheme 2:**
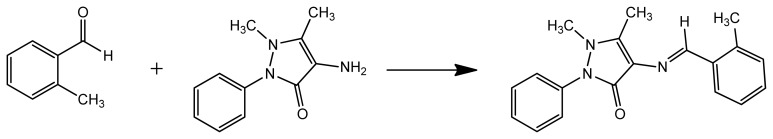
Synthesis of DMPO.

To synthesize the new corrosion inhibitor DMPO, the reaction sequence outlined in Scheme 2 was followed, starting from commercially available 4-aminoantipyrine. The synthesis was carried out by refluxing 4-aminoantipyrine with 2-methylbenzaldehyde in the presence of a few drops of acetic acid. The mechanism of this reaction followed the Schiff base mechanism.

**Scheme 3:**
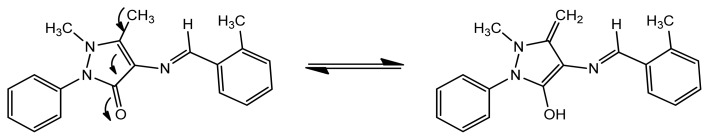
Tautomerization of DMPO.

The IR spectrum provided good evidence for the formation of the synthesized DMPO. In the IR spectrum of DMPO, the imine stretching frequency was observed at 1588.6 cm^−1^. The high value of the C=N wavenumber was due to the high conjugation (resonance effect) of the substituted double bonds, whereas the aromatic carbon-carbon double bond stretching appeared at 1569.4 cm^−1^. However, two types of tautomers, *i.e.*, amine and imine, could be expected from the DMPO structure (Scheme 3).

In the ^1^H-NMR spectrum of DMPO, a 1H singlet was observed at δ 9.712 ppm due to the imine proton.

### 2.2. Electrochemical

#### Electrochemical Impedance Spectroscopy (EIS) Measurements

**Figure 1:**
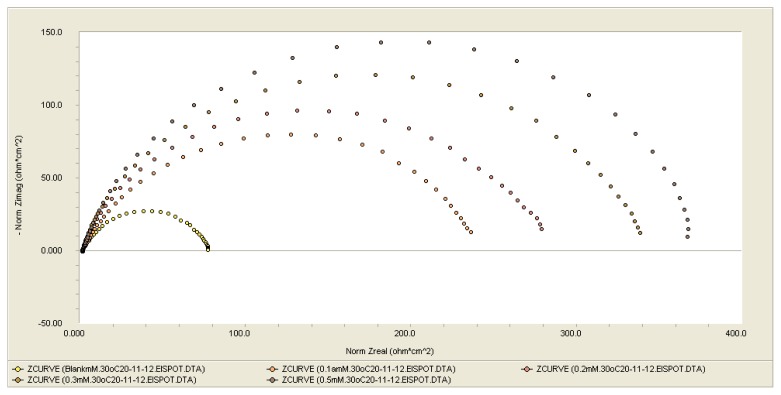
Nyquist plots for mild steel in 1.0 M HCl with DMPO at various concentrations.

The experimental results obtained from the EIS measurements for the corrosion of mild steel in the absence and presence of the inhibitor at 30 °C are summarized in Table 1. The impedance spectra for the mild steel samples in 1.0 M HCl in various concentrations of DMPO at 30 °C are presented as Nyquist plots in Figure 1. As shown in Figure 1, a considerable increase in the total substrate impedance was observed with increasing concentration of DMPO inhibitor added to the corrosive solution. In the impedance spectrum of the mild steel in the presence of DMPO, the Nyquist plots have two loops: one loop in the high frequency region (HF) and one loop at an intermediate frequency (MF), with slight inductive behavior at low frequencies (LF). The HF and MF loops were attributed to the electrode and the charge-transfer process, respectively. The inductive behavior observed in the LF region was attributed to either the relaxation of the adsorption of corrosion products or the adsorption of the inhibitor molecules on the mild steel surface in the acid solution in the absence or presence of inhibitor, respectively [21,22]. The inhibition efficiencies (IE%) were calculated from the charge transfer resistance using the following equation:

(1) $$\text{IE}(\%)=\frac{{\text{R}}_{\text{ct}}^{-}-{\text{R}}_{\text{ct}}}{{\text{R}}_{\text{ct}}^{-}}\times 100$$

where R_ct_^−^ and R_ct_ indicate the values of the charge transfer resistances in the presence or absence of inhibitor, respectively.

From Table 1, it can be observed that the R_ct_ and IE% values increase with increasing concentration.

In these cases, the parallel network charge-transfer resistance double-layer capacitance (R_ct_–C_dl_) is usually a poor approximation, especially for systems in which an efficient inhibitor is present. The corroding surface of the mild steel in 1.0 M HCl is expected to be inhomogeneous because of its roughness; therefore, the capacitance is presented through a constant phase element (CPE).

### 2.3. Polarization Measurements

The numerical values of the variations in corrosion current density (icorr), corrosion potential (Ecorr), anodic Tafel slope (βa), cathodic Tafel slope (βc), the degree of surface coverage (θ) and inhibition efficiency (IE%) with the concentrations of inhibitor DMPO are given in Table 1. The surface coverage (θ) is calculated thus [22]:

(2) $$\theta =i_{\text{corr}(\text{uninh})}-i_{\text{corr}(\text{inh})}/i_{\text{corr}(\text{uninh})}$$

where icorr(uninh) and icorr(inh) are the corrosion current densities in the absence and presence of the inhibitor, respectively. The inhibition efficiency (IE%) is given by

(3) IE%=θ×100

**Figure 2:**
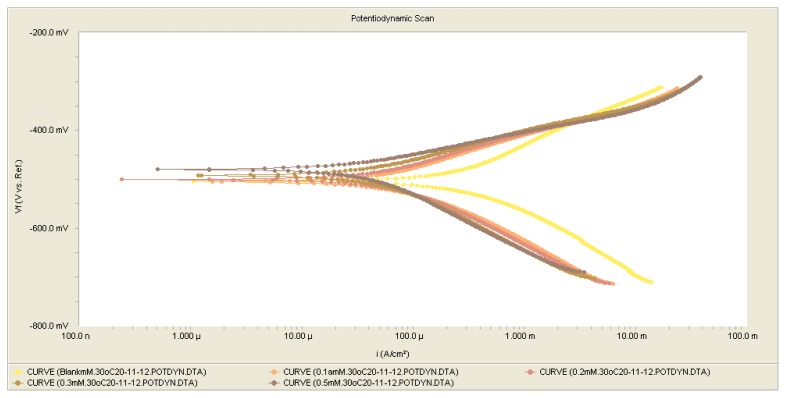
Potentiodynamic polarization curve for mild steel in 1.0 M HCl with various concentrations of DMPO at 30 °C.

The results also indicate that the inhibition efficiencies increased with the concentration of inhibitor. Such behavior can be interpreted on the basis that the inhibitor acts by adsorbing onto the metal surface. In acidic solutions, the anodic reaction of corrosion is the passage of metal ions from the metal surface into the solution, and the cathodic reaction is the discharge of hydrogen ions to produce hydrogen gas or to reduce oxygen. The inhibitor may affect either the anodic or the cathodic reaction, or both [23]. Because the anodic Tafel slope (βa) and cathodic Tafel slope (βc) of DMPO, were found to change with inhibitor concentration, the inhibitor had thus affected both of these reactions [24]. DMPO can thus be classified as an anodic- or cathodic-type inhibitor when the change in the Ecorr value is greater than 85 mV [25]. Because the largest displacement exhibited by DMPO was 40 mV at 30 °C (Table 2), it may be concluded that this molecule should be considered a mixed-type inhibitor, meaning that the addition of DMPO to a 1.0 M HCl solution both reduces the anodic dissolution of mild steel and retards the cathodic hydrogen evolution reaction. The polarization profile of mild steel in 1.0 M HCl at 30 °C in the presence and absence of DMPO is shown in Figure 2. The presence of increasing amounts of DMPO led to a decrease in both the cathodic and anodic current densities. Adsorption is the mechanism that is generally accepted to explain the inhibitory action of organic corrosion inhibitors. The adsorption of inhibitors can affect the corrosion rate in two ways: (i) by decreasing the available reaction area, *i.e.*, the so-called geometric blocking effect, and (ii) by modifying the activation energy of the cathodic and/or anodic reactions occurring in the inhibitor-free metal in the course of the inhibited corrosion process. It is a difficult task to determine which aspects of the inhibiting effect are connected to the geometric blocking action and which are connected to the energy effect. Theoretically, no shifts in Ecorr should be observed after the addition of the corrosion inhibitor if the geometric blocking effect is stronger than the energy effect [23].

#### 2.3.1. Open Circuit Potential (OCP) Measurements

**Figure 3:**
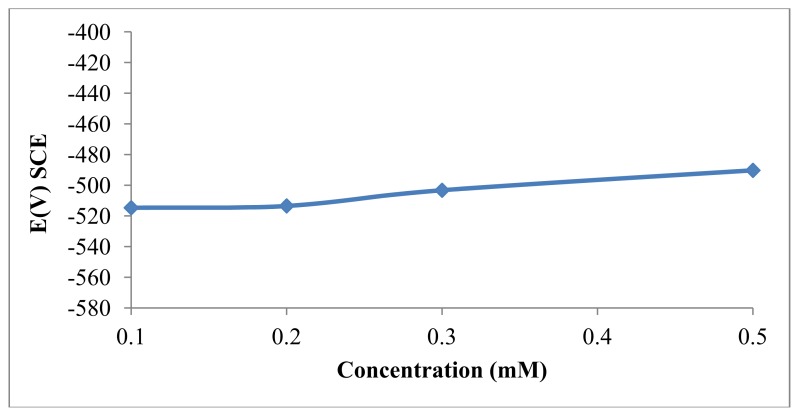
Open circuit potential (OCP) value for mild steel in an HCl solution with various concentrations of DMPO at 30 °C.

The OCP of mild steel was monitored in the presence of 0 mM, 0.1 mM, 0.2 mM, 0.3 mM and 0.5 mM DMPO. Figure 3 presents the effect of the presence of the DMPO inhibitor on the variation of the OCP of mild steel in 1.0 M HCl solutions. This preliminary result suggests that DMPO can retard both reactions under open circuit conditions, including the oxidation of the oxide-free iron and the discharge of the hydrogen ions to produce hydrogen gas on the surface of the mild steel [26,27].

#### 2.3.2. Electrochemical Frequency Modulation Measurement

Electrochemical frequency modulation (EFM) is a new electrochemical technique for determining the corrosion rate without preliminary knowledge of the Tafel constants. The main advantages of this technique include measuring the corrosion rate, Tafel parameters and causality factors in a single set of data. While using EFM, a potential perturbation signal composed of two sine waves is applied to any corroding specimen to obtain the current response. EFM has been used for different combinations of metals and electrolytes to accurately measure the corrosion parameters. EFM is related to the harmonic method of employing a lower amplitude (20 mV) sinusoidal perturbation signal but is composed of two sine waves instead of one. EFM is superior to the harmonic method in many aspects, including data validation, larger current response and insensitivity to harmonics in the perturbation signal.

The corrosion parameters, including the corrosion efficiency (E_EFM_%), the corrosion current density (μA/cm^2^), the Tafel constant and the causality factors (CF-2 and CF-3), with different concentrations of DMPO in 1.0 M HCl at a constant temperature of 30 °C are listed in Table 3.

#### 2.3.3. Quantum Chemical Calculations

Recently, density functional theory (DFT) has been used to analyze the characteristics of the inhibitor/surface mechanism and to describe the structural nature of the inhibitor in the corrosion process. Furthermore, DFT is considered a very useful technique to probe the inhibitor/surface interaction as well as to analyze the experimental data. This technique has been found to be successful in providing insights into the chemical reactivity and selectivity in terms of global parameters such as electronegativity (v), hardness (g) and softness (S), and local softness (sđ ~ r Þ) [28,29].

**Figure 4:**
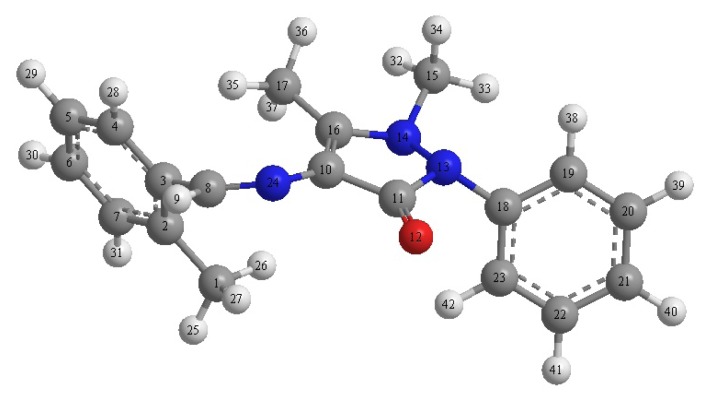
The 3D-structure of synthesized compound DMPO.

The design of the DMPO compound for use as a corrosion inhibitor was based on several factors. First, the molecule contains sulfur and nitrogen atoms (apart from the azomethine group) as active centers in addition to an azomethine group, which boasts biological functionality as an antibacterial and antifungal [30–32] agent. Second, DMPO can be easily synthesized and characterized [33,34]. The optimized molecular structure of the synthesized compound is shown in Figure 4.

The calculated quantum parameters of the HOMO, LUMO, band gap and dipole moment for DMPO are presented in Table 4.

Frontier orbital theory was useful in predicting the adsorption centers of the inhibitor molecule DMPO responsible for its interaction with surface metal atoms [35]. Terms involving the frontier molecular orbital could provide a dominative contribution because of the inverse dependence of stabilization energy on orbital energy difference. Excellent corrosion inhibitors are usually organic compounds that not only offer electrons to unoccupied orbitals of the metal but also accept free electrons from the metal [36].

A relationship between the corrosion inhibition efficiency of the synthesized compound with the orbital energies of the HOMO (E_HOMO_) and LUMO (E_LUMO_) as well as the dipole moment (μ) are shown in Table 4. As is clearly observed in the Table 4, the inhibition efficiency increases with an increase in E_HOMO_ values along with a decrease in E_LUMO_ values.

The E_HOMO_ is often associated with the electron donating ability of a molecule. Therefore, increasing values of E_HOMO_ indicate a higher tendency for the donation of electron(s) to the appropriate acceptor molecule with low energy and an empty molecular orbital. Increasing values of E_HOMO_ thus facilitate the adsorption of the inhibitor. Consequently, improving the transport process through the adsorbed layer would enhance the inhibition efficiency of the inhibitor. This finding can be explained as follows. E_LUMO_ indicates the ability of the molecule to accept electrons; therefore, a lower value of E_LUMO_ more clearly indicates that the molecule would accept electrons [37]. The dipole moment (μ) is an index that can also be used to predict the direction of a corrosion inhibition process. Dipole moment is the measure of polarity in a bond and is related to the distribution of electrons in a molecule. Although literature is inconsistent on the use of μ as a predictor of the direction of a corrosion inhibition reaction, it is generally agreed that the adsorption of polar compounds possessing high dipole moments on the metal surface should lead to better inhibition efficiency. The data obtained from the present study indicate that the DMPO inhibitor has the value of μ = 1.4655 and highest inhibition efficiency (85%). The dipole moment is another indicator of the electronic distribution within a molecule. Some authors state that the inhibition efficiency increases with increasing values of the dipole moment, which depends on the type and nature of molecules considered. However, there is a lack of agreement in the literature on the correlation between μ and %IE, as in some cases no significant relationship between these values has been identified [38,39].

The Mulliken charge distribution of DMPO is presented in Table 5. It has been reported that as the Mulliken charges of the adsorbed center become more negative, the atom more easily donates its electron to the unoccupied orbital of the metal [40]. It could be readily observed that nitrogen, oxygen and some carbon atoms have high charge densities. The regions of highest electron density are generally the sites to which electrophiles can attach [41]. Therefore, N, O and some C atoms are the active centers, which have the strongest ability to bond to the metal surface. Conversely, some carbon atoms carry positive charges, which are often sites where nucleophiles can attach. Therefore, DMPO can also accept electrons from Fe through these atoms. It has been reported that excellent corrosion inhibitors can not only offer electrons to unoccupied orbitals of the metal but also accept free electrons from the metal [42].

**Figure 5:**
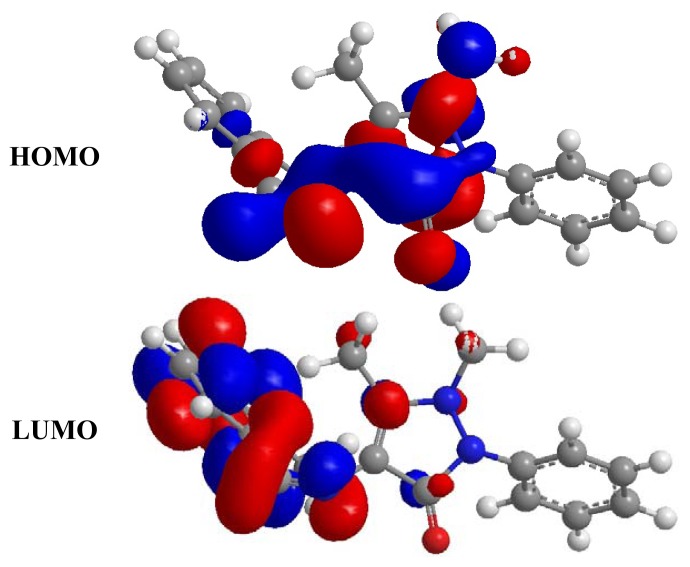
The HOMO and LUMO of DMPO.

According to the description of frontier orbital theory, HOMO (Figure 5) is often associated with the electron donating ability of an inhibitor molecule. High EHOMO values indicate that the molecule has a tendency to donate electrons to a metal with unoccupied molecule orbitals. ELUMO, conversely, indicates the ability of the molecule to accept electrons [43]. A lower value of ELUMO indicates an easier acceptance of electrons from a metal surface [44]. The gap between the LUMO and HOMO energy levels of inhibitor molecules is another important parameter. Low absolute values of the energy band gap (E = E_LUMO_ – E_HOMO_) mean good inhibition efficiency [45].

## 3. Experimental Section

All chemical**s** used were of reagent grade (supplied by Sigma-Aldrich Malaysia) and used as supplied without further purification. The FT-IR spectra were measured using a Thermo Scientific Model Nicolet 6700 spectrophotometer. NMR spectra were recorded on a Model AVANCE III 600 MHz spectrometer.

### 3.1. Synthesis of Corrosion Inhibitor DMPO

A solution of 2-methylbenzaldehyde (0.4 mmol) in 50 mL ethanol was refluxed with 4-aminoantipyrine (0.4 mmol) for 5 h. After cooling to room temperature, a solid mass was separated and recrystallized from ethanol in 87% yield. ^1^H-NMR (CDCl_3_): δ 9.712 (s, N=C–H), 6.983, 7.214, 7.307, 7.322, 7.418, 7.675, 7.711, 7.775, 7.876 (s, 1H, aromatic ring), δ 2.017 (s, 3H, CH_3_), 2.964 (s, 3H, CH_3_), δ 3.128 (s, 3H, CH_3_); IR: 3050.0, 3061.6 cm^−1^ (C–H, aromatic), 2910.7, 2945.9 and 2970.0 cm^−1^ (C–H, aliphatic), 1646.6 cm^−1^ (C=O), 1569.4 cm^−1^ (C=C); 1588.6 cm^−1^ (C=N, imine), 1484.4 cm^−1^ (C=C, aromatic); UV-Vis: 250 nm in acetonitrile.

### 3.2. Electrochemical Measurements

Mild steel specimens obtained from the Metal Samples Company were used as the working electrodes throughout the study. The composition (wt %) of the mild steel was as follows: Fe, 99.21; C, 0.21; Si, 0.38; P, 0.09; S, 0.05; Al, 0.01. The specimens were cleaned according to ASTM standard G1-03 [46,47]. Measurements were performed in aerated non-stirred 1.0 M hydrochloric acid solutions at 30 °C with a concentration range of 1.25 × 10^−4^ to 5 × 10^−4^ M DMPO corrosion inhibitor. Solutions were freshly prepared from analytical grade chemical reagents using distilled water. All measurements were performed in triplicate, and the average values were reported. Measurements were performed using a Gamry Instrument Potentiostat/Galvanostat/ZRA model. The DC105 and EIS300 software by Gamry were used for potentiodynamic scans and electrochemical impedance spectroscopy (EIS). The potentiodynamic current-potential curves were swept from −0.25 to +0.25 V_sce_ at a scan rate of 0.5 mV·s^−1^. All impedance data were fitted to appropriate equivalent circuits (EC) using Gamry Echem Analyst software. Experiments for electrochemical measurements were initiated approximately 30 min after the working electrode was immersed in the solution to stabilize the steady state potential.

### 3.3. Theory and Computational Detail

The molecular sketch of the synthesized compound was plotted using Gaussian 03, Revision C.01. All quantum chemical calculations were performed using Density Functional Theory (DFT). The following quantum chemical indices were calculated: the energy of the highest occupied molecular orbital (E_HOMO_), the energy of the lowest unoccupied molecular orbital (E_LUMO_) and dipole moment (μ).

## 4. Conclusions

In this study, a new 4-aminoantipyrine, *i.e.*, 1,5-dimethyl-4-((2-methylbenzylidene)amino)-2- phenyl-1*H*-pyrazol-3(2*H*)-one (DMPO), was sequentially synthesized and characterized using various spectroscopic methods. Changes in the electrochemical impedance spectroscopy (EIS), open circuit potential (OCP) and potentiodynamic polarization were used to study the corrosion inhibition of mild steel in 1.0 M HCl solutions at 30 °C using different concentrations of DMPO as an inhibitor. This compound exhibited excellent inhibition performance as a mixed-type inhibitor. In general, the acidic corrosion of mild steel was reduced upon the addition of an appropriate concentration of DMPO. The inhibition efficiencies obtained from the EIS data were comparable to those obtained from the polarization measurements in that they were greater for the inhibitory solution than those of the non-inhibitory solution. DMPO acts as an efficient corrosion inhibitor in 1.0 M hydrochloric acid and it exhibits a maximum inhibition efficiency of 87%. The adsorption of DMPO on a mild steel surface obeys the Langmuir adsorption isotherm. The correlation between the quantum chemical parameters and inhibition efficiencies of DMPO was investigated using DFT calculations. The inhibition efficiencies of the inhibitor are closely related to the quantum chemical parameters EHOMO, ELUMO and dipole moment. The fact that inhibition efficiency is increased with an increase in E_HOMO_ values and with a decrease in E_LUMO_ values has been established herein.

## Figures and Tables

**Table 1:** Electrochemical impedance spectroscopy (EIS) parameters for mild steel in 1.0 M HCl with various concentrations of 1,5-dimethyl-4-((2-methylbenzylidene)amino)-2- phenyl-1*H*-pyrazol-3(2*H*)-one (DMPO) at 30 °C.

Conc., 1 × 10^−3^M	R_s_, ohm cm^2^	R_ct_, ohm cm^2^	CPE_dl_ (Y0X10^−5^), ohm^−1^cm^−2^S^n^	IE (%)
0	–	77	–	0
0.1	1.55	239	39.4	71.08
0.2	1.56	259	22.2	70
0.3	1.63	328	17.7	77
0.5	1.73	376	23.3	80

**Table 2:** Polarization parameters for mild steel in 1.0 M HCl with different concentrations of DMPO at 30 °C.

Conc., 1 × 10^−3^M	i_corr_ (μA cm^−2^)	−E_corr_ (mV *vs.* SCE)	β_a_ (V dec^−1^)	β_c_ (V dec^−1^)	IE%
0	298	504	0.119	0.121	0
0.1	60	505	0.07	0.10	79.860
0.2	49	500	0.06	0.10	83.550
0.3	40.5	492	0.06	0.11	86.410
0.5	39.6	479	0.06	0.12	87.700

**Table 3:** Electrochemical frequency modulation (EFM) parameters for mild steel in 1.0 M HCl with various concentrations of DMPO at 30 °C.

Conc, mM	*i*_corr_, (μA·cm^−2^)	β_a_, (V·dec^−1^)	β_c_, (V·dec^−1^)	CR mmpy	IE (%)
0	189.8	24.26e-3	27.00e-3	4.89	0
0.1	501.9	96.88e-3	152.5e-3	1.295	80
0.2	478.5	66.90e-3	173.2e-3	1.234	83
0.3	388.1	105.4e-3	152.4e-3	1.001	86
0.5	301.6	106.9e-3	120.8e-3	0.777	87

**Table 4:** Molecular properties of the optimized synthesized compound.

Comp.	HOMO eV	LUMO eV	Band gap	Dipole moment	Total Energy	Conc. (mM)	IE_Exp%_	IE_Theo%_
DMPO	−8.051	−1.593	−6.458	1.4655	54.2342 Kcal/Mol	0.1	80	67.30
	−8.051	−1.593	−6.458	1.4655	54.2342 Kcal/Mol	0.5	87	84.26

**Table 5:** Mulliken charge of the DMPO molecule.

Atom	Charge	Atom	Charge	Atom	Charge	Atom	Charge
C(1)	−0.1893	C(11)	0.3262	C(21)	−0.1205	H(31)	0.1352
C(2)	−0.0376	O(12)	−0.2802	C(22)	−0.1268	H(32)	0.0969
C(3)	−0.1087	N(13)	−0.1996	C(23)	−0.1147	H(33)	0.1058
C(4)	−0.1179	O(14)	−0.1130	N(24)	−0.1022	H(34)	0.0774
C(5)	−0.1377	C(15)	−0.1202	H(25)	0.0865	H(35)	0.1126
C(6)	−0.1181	C(16)	0.0037	H(26)	0.0922	H(36)	0.0946
C(7)	−0.1366	C(17)	−0.1875	H(27)	0.0972	H(37)	0.1073
C(8)	−0.0139	C(18)	0.0272	H(28)	0.1370	H(38)	0.1420
H(9)	0.1550	C(19)	−0.1048	H(29)	0.1351	H(39)	0.1344
C(10)	−0.1499	C(20)	−0.1332	H(30)	0.1344	H(40)	0.1341
